# Advanced methodology for damping characterization of the human tympanic membrane using high-speed digital holography and STFT analysis

**DOI:** 10.1038/s41598-026-50633-9

**Published:** 2026-05-05

**Authors:** L. F. Caminos, J. Garcia-Manrique, Jeffrey T. Cheng, A. Gonzalez-Herrera

**Affiliations:** 1 Department of Civil Engineering, Materials and Manufacturing, School of Engineering, University of Malaga, Málaga, Spain; 2 Eaton-Peabody Laboratory, Massachusetts Eye and Ear, Boston, MA USA; 3 Department of Otolaryngology–Head and Neck Surgery, Harvard Medical School, Boston, MA USA

**Keywords:** Tympanic membrane, damping, High-speed digital holographic, Short-Time Fourier Transform, Engineering, Optics and photonics, Physics

## Abstract

This study presents an experimental methodology for estimating frequency-dependent damping in the human tympanic membrane (TM) using full-field time-domain holographic measurements and Short-Time Fourier Transform (STFT) analysis. Although damping plays a critical role in middle-ear mechanics, its experimental estimation remains challenging, with reported values exhibiting substantial variability. A high-speed digital holography (HDH) system is employed to capture transient displacement fields of the TM surface from cadaveric human temporal bones subjected to acoustic click excitation. The proposed approach enables the analysis of damping as a function of frequency by isolating free-vibration decay in the time–frequency domain, overcoming limitations of conventional time-domain techniques in systems with multiple overlapping modes. The results reveal a clear frequency dependence of effective system-level damping and its spatial variation across the membrane, as well as the influence of ossicular chain loading. This method offers a practical framework for extracting damping information from HDH experiments. The methodology provides robust local effective damping values relevant for improving the calibration and validation of finite element models of middle-ear mechanics.

## Introduction

 The human middle ear (ME), comprising the tympanic membrane (TM), ossicular chain (OC), ligaments, and tendons, can be modeled as a multi-degree-of-freedom (MDOF) vibratory system using finite element method (FEM), which has been extensively used to investigate and analyze the vibrational phenomena in the auditory system through governing Eqs^1–7^. Accurate results, however, require a comprehensive understanding of the geometry and mechanical properties of all relevant components. The TM is a key component in the dynamic response of the human auditory system^8^. It converts incident acoustic energy into mechanical vibrations, which are subsequently transmitted to the OC and the cochlea, where the signal is transformed into neural impulses perceived as sound.

In structural dynamics, damping is the mechanism through which a system dissipates energy while in motion. It acts as a force opposing movement, gradually decreasing the amplitude of vibrations over time. Damping remains one of the least understood aspects of vibration analysis, as no universal mathematical model has been established. This is primarily due to uncertainties regarding the state variables influencing damping forces. Consequently, multiple mathematical models exist to account for different energy dissipation mechanisms but require experimental validation.

The effective damping of the ME arises from several distinct mechanisms. Structural viscoelastic damping of the TM and soft tissues dissipates energy internally and influences frequency response characteristics, as shown by finite element analyses incorporating tissue viscoelastic properties^9–11^.

Acoustic damping associated with the tympanic cavity is caused by viscous and thermal losses in the air volume, as well as by acoustic radiation toward connected anatomical structures such as the Eustachian tube and mastoid air cells, which introduce a frequency-dependent resistive effect on the membrane, particularly at low frequencies^12,13^.

Fluid–structure interaction between vibrating structures and surrounding air contributes further dynamic resistance in coupled acoustic–structural systems^14,15^. Moreover, the cochlear load imposes a dissipative impedance on the ossicular chain, and middle-ear muscle reflexes can modulate damping actively under high-intensity stimulation^16,17^.

Together, these contributions determine the effective damping observed experimentally, highlighting the importance of distinguishing structural and acoustically induced effects for accurate interpretation of measurements and the development of reliable finite element models of middle-ear sound transmission, including applications in prosthesis design and pathology diagnosis.

The high sensitivity of the dynamic response of the human ME to changes in the mechanical, physical, and geometric properties of the TM has been extensively studied in previous research^18–24^. Specifically, the damping of the TM exerts a particularly significant influence on sound transmission.

However, estimating TM damping requires methodologies capable of isolating its effects within the complex, multi-material ME system. Although substantial progress has been made in this area^2,7,10,22,25–29^ due to the improvement in experimental techniques and computational tools, there is still no universally accepted approach for TM damping estimation.

The high-speed digital holographic (HDH) system is an advanced technology designed to capture three-dimensional images of vibrating structures with high temporal resolution. This system utilizes a high-speed camera and an interferometer to record digital holograms, which are interference patterns created by the diffracted light from the object and a coherent reference wave. The specifics of this technique and its application in measuring displacements on the TM surface are detailed in^28–32^.

The Short-Time Fourier Transform (STFT) is a key tool in analyzing non-stationary signals, offering a joint representation in the time-frequency domain. This technique is widely applied across various fields, including audio and music processing, electric machine diagnostics, biomedical signal processing, as well as communications and radar^33–35^. The STFT has been used for vibration analysis, damage detection, and structural integrity monitoring in dynamic systems. It provides time-frequency analysis of non-stationary responses and helps identify changes in modal frequencies, damping, and mode shapes for structural health monitoring^36–42^. Its capabilities make STFT a promising tool for TM damping estimation.

This work presents a methodology for estimating TM damping based on time-domain experiments performed on a cadaveric human temporal bone. HDH is used to measure acoustically induced transient displacements of the TM surface, which are analyzed using STFT. While traditional linear, Rayleigh, and viscoelastic models usually treat damping as a material property or as a global/modal property, the present work aims to characterize the effective response distribution obtained experimentally from the coupled middle ear system.

Section "Background on estimation of TM damping using experimental and FEM methology." reviews TM damping estimation methods combining FEM and experimental validation. Section  "Theoretical basis of the stft applied to transient HDH data" describes the methodology used, the calculation of STFT and the determination of damping from them. Section  “Methods” details the proposed damping estimation procedure. Results are provided in Sect. “Results” followed by a discussion Sect. “Discussion” and finally conclusions in Sect.  “Conclusion”.

## Background on estimation of TM damping using experimental and FEM methology

This section provides a brief overview of the evolution of damping estimation in the human TM through FEM and its experimental validation.

To date, damping estimation can be classified into four stages, based on the assumptions concerning the material properties of the membrane, the geometric accuracy, and the experimental methodologies used for both damping characterization and model validation.

The first stage is characterized by the incorporation of a damping ratio (ξ) into the dynamic modeling of TM. In this procedure, the membrane is assumed to behave as a linear, elastic, and isotropic material, with its geometry reconstructed based on anatomical data reported in the literature.

The initial studies by Wada et al.^18^ estimated a damping ratio of $\:{\xi\:}_{E}=0.126$ for the TM. This value was derived from empirical data previously obtained by the same research group^2,43^. The damping ratio was estimated using eight temporal bone specimens obtained from recent autopsies and a custom-designed device capable of detecting changes of sound pressure level (SPL) applied to the TM across a frequency range of 0.1 to 2.0 kHz. The estimation was based on a theoretical model incorporating acoustic impedance and the dissipation of mechanical energy.

The second stage involved advancements in both geometric model accuracy and experimental methodology. Anatomical models were now based on data obtained through histological sectioning, micro-CT, or optical imaging techniques, and laser Doppler vibrometry (LDV) became widely used as an experimental tool. Researchers adopted the Rayleigh approximation as the standard approach to model the damping behavior of the TM, ligaments, and tendons in FEM simulations.

Koike et al.^3,44^ modeled the damping matrix [C] in FEM as the sum of two components: [C] = α[M] + β[K], where α and β represent the mass-proportional and stiffness-proportional damping coefficients, respectively. The estimated values were α = 260 s⁻¹ and β = 3.7 × 10⁻⁵ s, obtained by calibrating the numerical model to match the damping ratio used in earlier studies^2,18^.

Subsequent work by Sun, Gan, et al.^5,7,20,26,45^ combined LDV with FEM. In^26^, this methodology was applied to analyze the transfer function of the human middle ear by measuring vibrations at the TM, the incudostapedial joint, and the stapes footplate. Pure tone acoustic stimuli were applied at frequencies ranging from 0.1 to 10 kHz. The vibratory response was measured by detecting frequency shifts in the reflected laser beam at each point, and the resulting data were used to compute displacements as a function of frequency to derive the middle ear’s transfer function. Material characterization during this stage was limited to parameter fitting through FEM model calibration using engineering optimization techniques. In particular, damping coefficients were tuned to α = 0 s⁻¹ and β = 0.0001 s^7^, and later to α = 0 s⁻¹ and β = 0.75 × 10⁻⁴ s^5^.

The third stage represents a significant shift in the approach to damping. The TM material is considered viscoelastic^10,21,27^, enabled by new devices capable of measuring its response under dynamic loading conditions. TM damping is calculated using the loss factor η, defined as the ratio of the loss modulus E″(f) to the storage modulus E′(f).

A key study from this stage is that of De Greef et al.^22^, which evaluates the performance of two linear viscoelastic Maxwell models by comparing their simulated umbo and TM surface displacements with experimental data obtained via stroboscopic holography.

The first model is based on experimental data from Zhang et al.^10^, resulting in a loss factor that increases logarithmically from 1% at 20 Hz to 13% at 20 kHz. The second model uses Maxwell parameters derived from the results of Aernouts et al.^21^, maintaining a constant loss factor of 7.8% across all frequencies.

The findings show that the viscoelastic model more accurately captures the tissue’s dynamic response across a broad frequency range and better reflects the actual behavior of the TM within the audible spectrum. In contrast, Rayleigh based models produce excessive damping values at high frequencies and are unsuitable for FEM applications.

In recent years, HDH has emerged as a powerful tool for investigating the biomechanics of TM. Unlike stroboscopic holography, which is restricted to steady-state periodic excitation, HDH captures the time-resolved vibration field of the tympanic membrane, enabling analysis over a wide frequency range and direct estimation of damping variations with frequency.

Studies by Razavi et al.^30,46^, Tang et al.^29^, and Garcia-Manrique et al.^28^ using this technique have provided detailed insights into TM vibration patterns and material characterization. HDH enabled the measurement of TM displacements at over 100,000 points, allowing for a highly detailed assessment of its vibratory behavior.

In the context of damping estimation, Tang et al.^29^ analyzed transient responses of the TM induced by acoustic clicks from eight fresh human temporal bones. The frequency response function (FRF) and impulse response function (IRF) were employed to describe TM’s transient response to an impulsive acoustic stimulus. By assuming the dominant frequency determined from the FRF is the natural frequency of the TM, and estimating the decay time of the TM from IRF, the damping ratios (ξ) varied across the TM surface up to about 4 kHz were derived. For frequencies ranging from 0.5 to 4 kHz, ξ ranged from 0.6 to 0.8 at dominant frequencies below 700 Hz, and from 0.05 to 0.3 at frequencies above 2.1 kHz. In addition to confirming the frequency dependence of damping, the study also demonstrated its spatial variation across the TM surface. These findings were subsequently used to support the experimental validation of a transient middle ear model in^28^.

Despite these advances in experimental measurement techniques and numerical models, the damping phenomenon of the TM remains among the least understood ME mechanical properties. Numerical validation in^28^ and obtained results in^29^ still presents uncertainties and inter-specimen variability. The methodology presented in this work represents a significant improvement over previous procedures and is expected to reduce, where present, variability associated with signal processing and data interpretation. It will also improve ME simulation accuracy.

## Theoretical basis of the stft applied to transient hdh data

The STFT is a widely used method for analyzing non-stationary signals^47,48^, as it provides an effective time-frequency representation for many types of signals. To determine which frequencies are present at a specific time, the signal is divided into small segments around that time. Each segment is then analyzed using the Fast Fourier Transform (FFT), while the rest of the signal is ignored. STFT is based on FFT of a short portion of signal sampled by a moving window. It is particularly useful for studying the dynamic behavior of TM, capturing temporal changes due to damping.

A brief description of the procedure used to determine damping by combining HDH and STFT is presented in this section.

The HDH provides time-resolved measurements of the displacement amplitude of the tympanic membrane in a matrix of points. We will denote this time displacement function as A(x, y,t), where x and y are the position of each point. We will apply STFT to each time signal, obtaining a function in terms of frequency ($\:{\omega\:}_{k})$ of displacement for each window applied at time *t*_*p*_. It will be denoted as $\:U\left(x,y,{t}_{p},{\omega\:}_{k}\right)$.

The signal A(x, y,t) is divided into overlapping windows of n-samples and multiplied by a Hanning window (Eq. 1).

1 $$\:w\left(m\right)=\frac{1}{2}\left[1-\mathrm{cos}\left(\frac{2\pi\:m}{n-1}\right)\right],m=0,\dots\:,n-1$$

where:

$\:w\left(m\right)$= Hanning window applied to each segment, with $\:m=\mathrm{0,1},\dots\:,n-1$.

### $\:n$

number of samples STFT window.

The STFT of each windowed segment is:

2 $$\:U(x,y,{t}_{p},{\omega\:}_{k})=\sum\:_{m=0}^{n-1}A(x,y,{t}_{p}+m{\Delta\:}t\left){\hspace{0.17em}}w\right(m){\hspace{0.17em}}{e}^{-i{\omega\:}_{k}m{\Delta\:}t}$$

with:

3 $$\:{\omega\:}_{k}=\frac{2\pi\:k}{n{\hspace{0.17em}}{\Delta\:}t}$$

where:

$\:U(x,y,z,{t}_{p},{\omega\:}_{k})$= STFT of the displacement amplitude at location (*x*,* y*), window $\:{t}_{p}$, and frequency $\:{\omega\:}_{k}$.

$\:{t}_{p}$= initial time of each analysis window.

$\:{\omega\:}_{k}$= angular frequency of the $\:k$-th STFT component.

$\:{\Delta\:}t=\:$time sampling interval of HDH measurements.

Once we have $\:U\left(x,y,{t}_{p},{\omega\:}_{k}\right)$ along time (*t*_*p*_), we can evaluate the decay of the signal for each frequency.

For a lightly damped linear single degree of freedom system with zero phase, the amplitude at a given frequency decays exponentially following this expression:

4 $$\:A\left(t\right)={A}_{0}{\hspace{0.17em}}{e}^{-\xi\:{\omega\:}_{n}t}\mathrm{cos}\left({\omega\:}_{d}t\right)$$

where:

$$\:\xi\:\:$$= damping ratio.

$$\:{\omega\:}_{n}$$= natural frequency.

### $\:{\omega\:}_{d}={\omega\:}_{n}\sqrt{1-{\xi\:}^{2}}$

damped natural frequency.

$\:{A}_{0}$ = initial amplitude of vibration at $\:t=0$.

Damping ratio $\:\xi\:$ is defined as the ratio among damping (c) and critical damping (c_c_ = $\:2\sqrt{m\cdot k})$ considering a single degree of freedom system. Damping ratio $\:\xi\:$ is normally provided in terms of percentage.

If we evaluate the amplitude of the time signal at two different time $\:{A(t}_{1})$ and $\:{A(t}_{2})$ we can obtain the expression for the logarithmic decrement $\:\delta\:\:$as:

5 $$\:{\updelta\:}=\mathrm{ln}\left(\frac{A\left({t}_{2}\right)}{A\left({t}_{1}\right)}\right)=-\xi\:{\omega\:}_{n}\left({t}_{2}-{t}_{1}\right)=-\xi\:{\omega\:}_{n}{\Delta\:}t$$

So, we can obtain damping for a given natural frequency. The problem is that we cannot determine properly the logarithmic decrement directly from A(x, y,t), so we must resort to the STFT function $\:U\left(x,y,{t}_{p},{\omega\:}_{k}\right)$.

In this case, fitting a linear equation for the function $\:ln\left|U\left(x,y,{t}_{p},{\omega\:}_{k}\right)\right|$ in terms of *t*_*p*_, we can obtain the slope corresponding to the product $\:{\xi\:}_{k}{\omega\:}_{k}$ and so the frequency-dependent damping ratio function $\:\xi\:\left({x,y,\omega\:}_{k}\right)$= $\:{\xi\:}_{k}$. Within this approach, we can average the function $\:{\xi\:}_{k}$ for different areas (in terms of x, y) to obtain a representative value for each region and frequency, as demonstrated in Sect.  “Results”.

It should be noted that the averaging process is not intended to represent a local material property, but rather to obtain a representative effective damping value at the system level, consistent with the global dynamic response of the middle ear. This strategy is particularly relevant in the context of finite element model calibration, where simplified damping descriptions are commonly employed.

## Methods

The estimation of TM damping was performed using STFT-based time-frequency analysis of displacement data of the TM acquired with HDH from a human temporal bone specimen (TB-03). The details of our measurement techniques and experimental procedures can be found in our previous studies^29,32,46^, but are briefly described below.

### Data acquisition using HDH

**Fig. 1:**
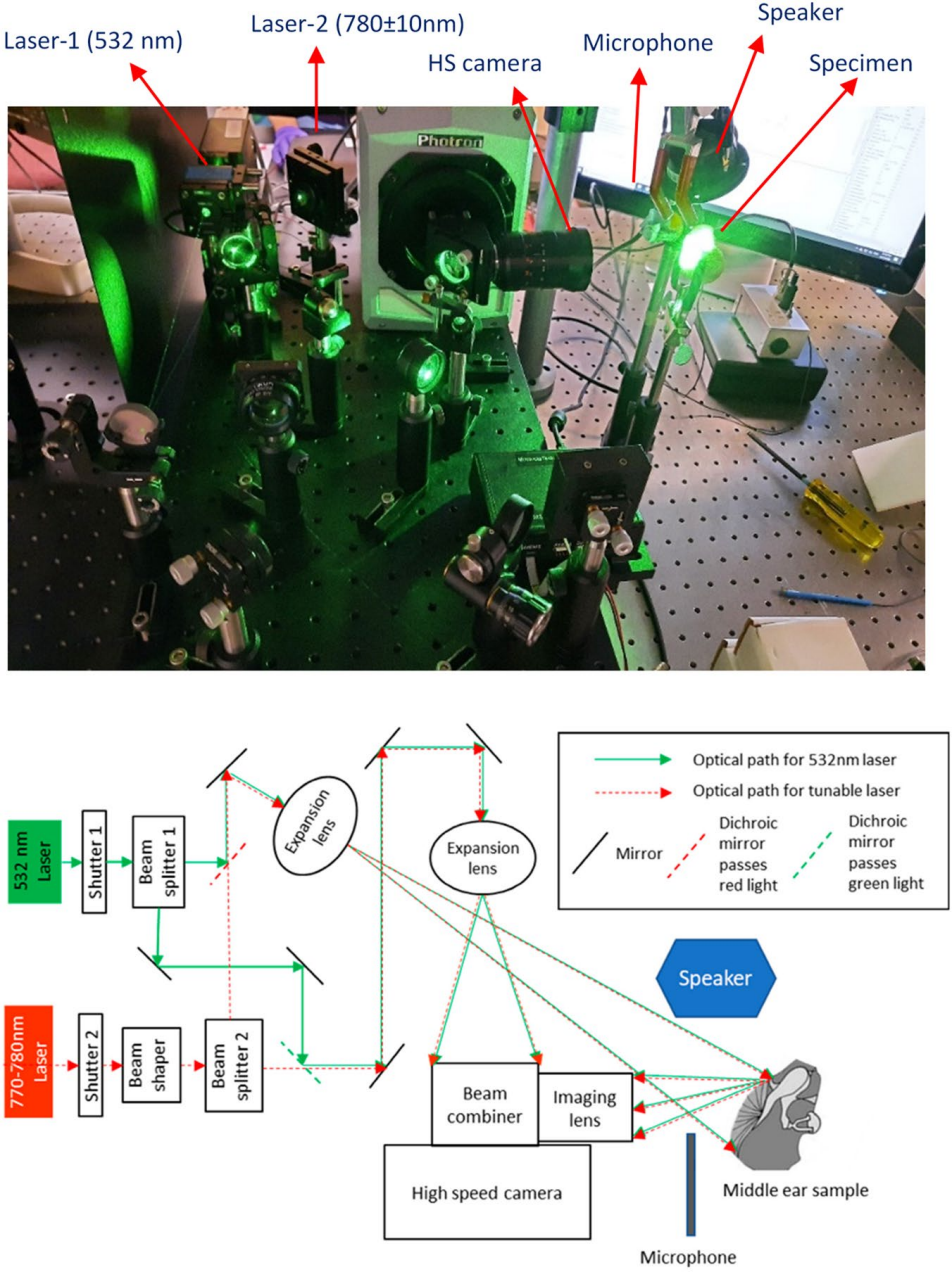
Experimental setup and Scheme of the HDH system^32^.

HDH was used to measure transient vibrations of fresh post-mortem human TM induced by acoustic clicks (Fig.1). The acoustic click used in our study is generated by a speaker driven by a 50 µs square electrical pulse. The cartilaginous and bony ear canal were removed to expose the TM to the HDH camera, the effect of removing the ear canal on the TM motion is negligible at frequencies below 10 kHz^49^.

The system captured displacement and shape at over 100,000 points per TM surface, with a temporal resolution below 20 µs and a displacement resolution of approximately 10 nm. Lateral resolution for shape measurement was approximately 100 μm. Two lasers operated on the same optical path: a 532 nm laser for displacement and a tunable 770–780 nm laser for shape measurement. The TM’s response to a 50 µs square wave acoustic click was recorded. A Knowles FG-23,329 microphone placed nearby the TM annulus captured the stimulus profile.

Displacement measurements were obtained via correlation interferometry, mapping full-field TM motion at 67,200 fps. Shape was measured using Multiple Wavelength Holographic Interferometry (MWHI). MWHI also provided surface normal vectors, enabling precise displacement direction analysis.

The microphone recorded for 25 ms, with a 3.65 ms delay between the speaker’s trigger signal and the sound wave reaching the microphone.

### Methodology description and illustrative example

This section presents an illustrative example of the methodology applied to a single point. All parameter selections used in the final analysis are described in Sect.  “Results”.

The methodology adopted for the estimation of TM damping builds upon previous work^29^ where an exponentially decaying function was fitted to the IRF. In the present study, we present a new framework to derive the damping of the TM with improved results.

The method is based on the concept of logarithmic decrement for a single-degree-of-freedom system under free vibration. It consists of evaluating the ratio between signal amplitudes at different time instants and fitting an exponential decay model to estimate the damping coefficient. When the displacement magnitude is expressed on a logarithmic scale, this relationship becomes linear.

However, in the present experimental data, the time signal shows a complex combination of multiples natural frequencies, making it impossible to distinguish the decay at each frequency. To overcome this limitation, a STFT analysis is applied to the signal. This approach enables the decomposition of the response, allowing the decay of each frequency contribution to be evaluated independently. The resulting estimates can be interpreted as a local effective damping, rather than as theoretical modal damping ratios of a linear MDOF system.

In this section, the procedure is illustrated using a single point on the TM surface. For clarity, some technical aspects have been simplified to facilitate interpretation. The results obtained are representative, although further refinement is achieved through the optimized parameter described in next sections.

**Fig. 2:**
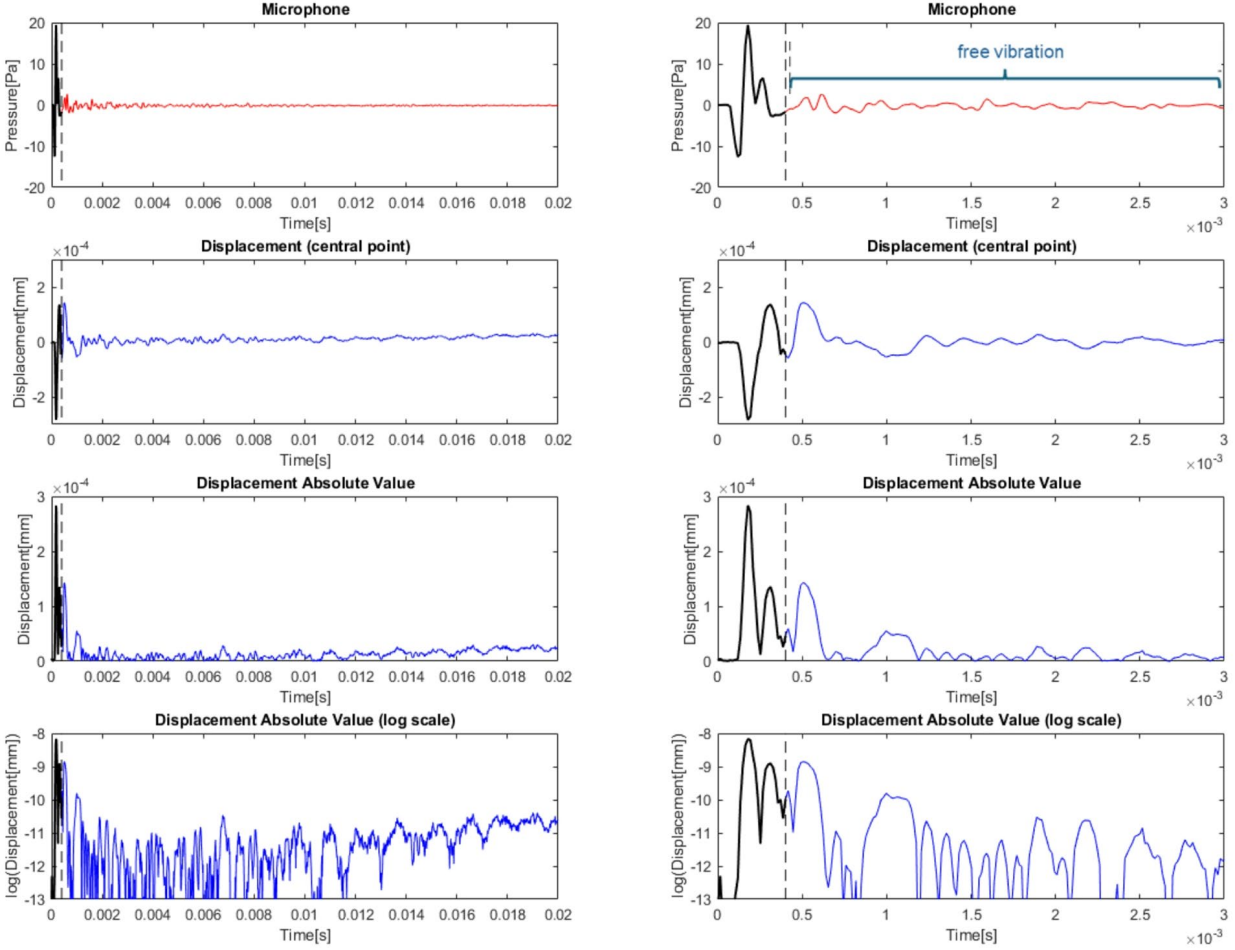
Microphone pressure and displacement results corresponding to the central point of the membrane. Time scale enlarged on the second column.

The displacement on the entire surface of the TM in the time domain is extracted from holographic reconstruction. Figure 2 presents the signal captured by the microphone and the displacement results corresponding to the central point of the membrane. This arbitrary central membrane point was chosen to illustrate key aspects of the data analysis. Later the methodology proposed will be extended to the rest of the points on the TM surface.

The microphone signal distinguishes the first incident wave (before t = 0.4 ms, top row in Fig. 2, black curve) and posterior waves corresponding to the sound reflected from the TM motion (after t = 0.4 ms, red curve in Fig. 2 top row). Membrane response exhibits two different behaviors. First a forced vibration phase (second row in Fig. 2, black curve), due to the incident wave, followed by free vibration (blue curve in Fig. 2 second row) where damping estimation is possible. These periods of time have been highlighted in Fig. 2.

The third row of Fig. 2 plots the absolute value of the displacement from the row above. If we plot the displacement signal on a logarithmic scale of the absolute value of the displacement, we can observe clearly both behaviors (forced and free vibration). In logarithmic scale, damping would be directly related to the slope of the decreasing line obtained during the free vibration period. The problem is that the displacement signal is the combination of several vibration modes moving and damping simultaneously. So, we cannot fit one single function, and we must separate the signal at different frequencies.

This is clearly observed in the logarithmic scale figure where we can see how the signal, apparently, does not die out to zero. This effect is due to the presence of very low frequency motion. This motion is related in this test to the supporting system; it is not of interest to us but makes impossible to evaluate damping from the temporal signal directly.

STFT techniques will be used to evaluate the decreasing magnitude with time separately for each frequency. It is applied to the portion of the signal corresponding to free vibration behavior.

**Fig. 3:**
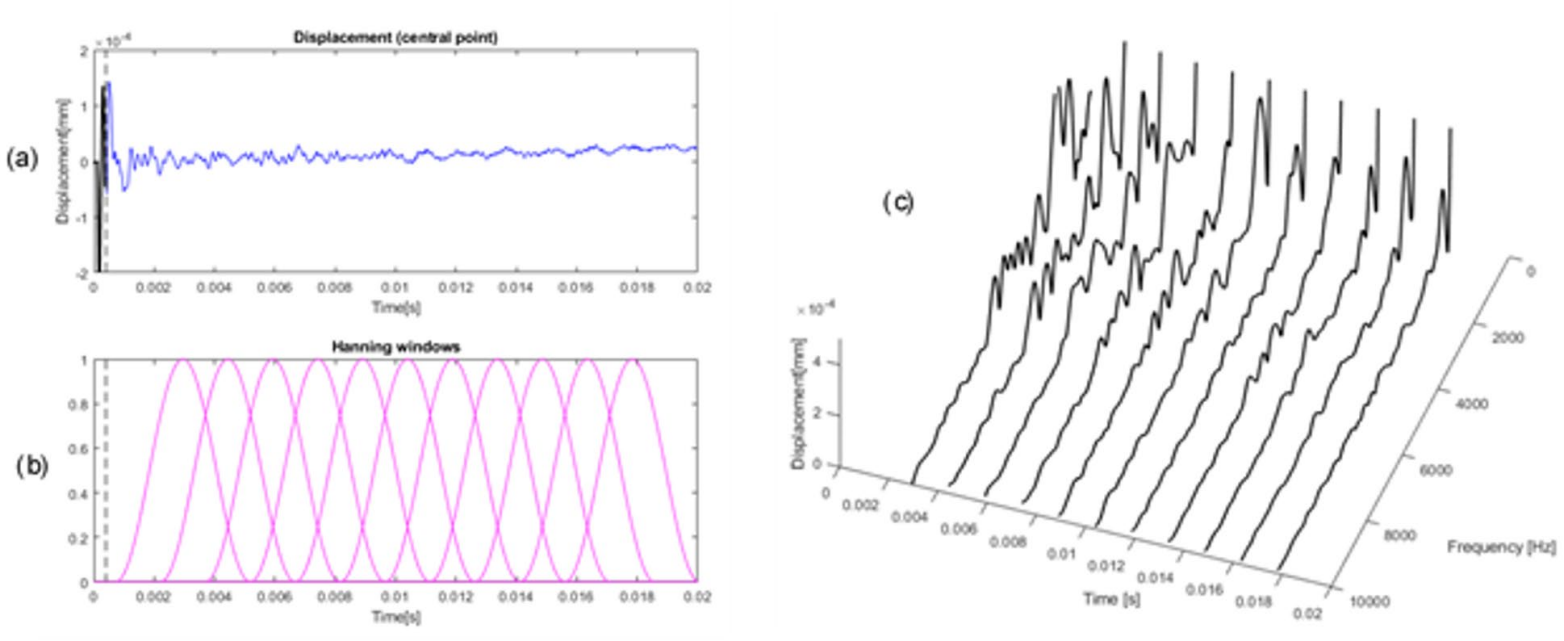
Displacement signal (**a**), windows applied (**b**) and FFT in time (**c**).

The signal is segmented into overlapping windows, and the FFT is applied to each window to generate a time–frequency representation. Figure 3 schematically illustrates this process. Several parameters influence the frequency detection capability, including the choice of window function, window length, and the number of windows. These parameters are discussed in detail in the next section. For illustrative purposes, a limited number of 11 windows is used in this example. Despite this reduced number, the results remain representative. The first window is positioned at a time where the effect of forced vibration is no longer present.

In Fig. 3, the displacement signal is shown in panel (a), along with the applied time windows in panel (b). In this case, Hanning windows with a length of 300 points (T = 4.5 ms, fₘ_i_ₙ = 336 Hz, considering fₘ_i_ₙ =1.5/T)) are applied. For the central time of each window, the corresponding FFT is computed. These FFTs are plotted over time in Fig. 3(c), providing a time-frequency representation of the signal. Several important aspects can be identified in this graph.

First, the figure reveals the presence of low-frequency components that do not correspond to the expected TM frequencies. These low frequencies are induced by the rigid-body motion of the ear’s supporting structure. Furthermore, as previously mentioned, the time-domain signal exhibits an unexpected increase in magnitude as the damping progresses. This behavior is attributed to the influence of the low-frequency background signal, which clearly does not originate from the TM itself and can be ignored. It is crucial to distinguish such experimental artifacts from noise in order to prevent the loss of critical high-frequency information during filtering or normalization procedures.

Second, focusing on the range of frequency of interest, that is above the minimum frequency imposed by the parameter of STFT methodology (in this case fₘ_i_ₙ = 336 Hz), a clear decreasing trend in the magnitude response over time can be observed. This decay corresponds to the damping of the system and can be quantified by fitting an exponential decay function to the magnitude envelope. So, we can estimate damping at each frequency.

**Fig. 4:**
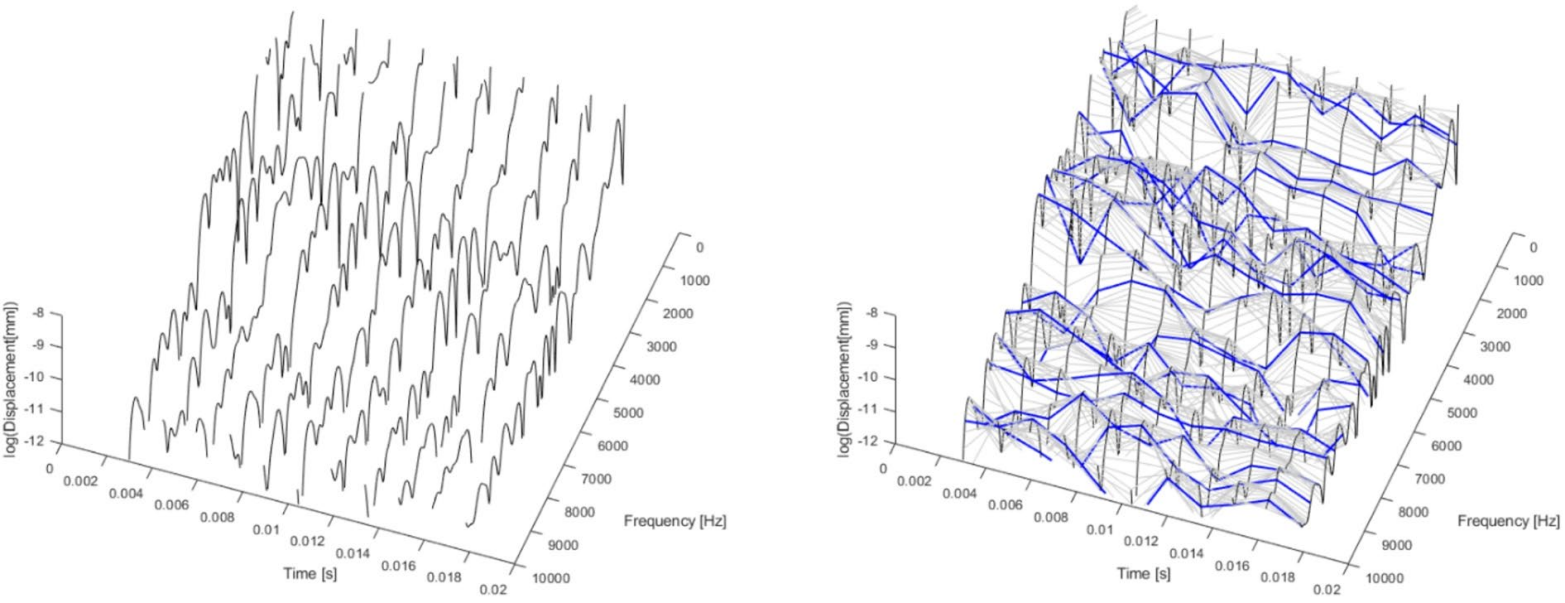
Displacement FFT in time with logarithmic scale.

This analysis is illustrated in Fig. 4, which again presents partial data acquired at the center of the membrane. In this figure, the FFT magnitudes are plotted on a logarithmic scale, showing the evolution of spectral slopes over time for each analyzed frequency. This information provides the foundation for damping estimation. To quantify the damping coefficient, an exponential decay model of the form $\:A\left(t\right)={A}_{0}{e}^{-\xi\:{w}_{n}t}\:$is used to fit the data, where $\:\xi\:$ is the damping ratio and $\:{w}_{n}$ is the natural frequency in rad/s (see Sect.  "Theoretical basis of the STFT applied to transient HDH data " for details).

As we do not know the natural frequencies, we will do this procedure for the whole range of frequencies (from 0 to 10 kHz every 49.4 Hz) independently from the fact that it is a natural frequency of the system or not. As it will be seen with the results obtained, those frequencies where there is no influence from closer natural frequencies provide no valid results. This point is a key factor to interpret the result obtained.

The logarithmic decrement is then calculated by analyzing successive peaks in the time response. On a logarithmic scale, the product $\:\xi\:{w}_{n}$ corresponds to the slope of a decay curve. In Fig. 4.b, lines connecting FFT values over time for different frequencies have been added in order to illustrate the fitting process. It is important to note that, for illustrative purposes, these figures correspond to results obtained with a relatively small number of windows, and therefore FFTs. Increasing the number of windows improves the accuracy and robustness of the damping estimation. This is done for the whole data in next section.

**Fig. 5:**
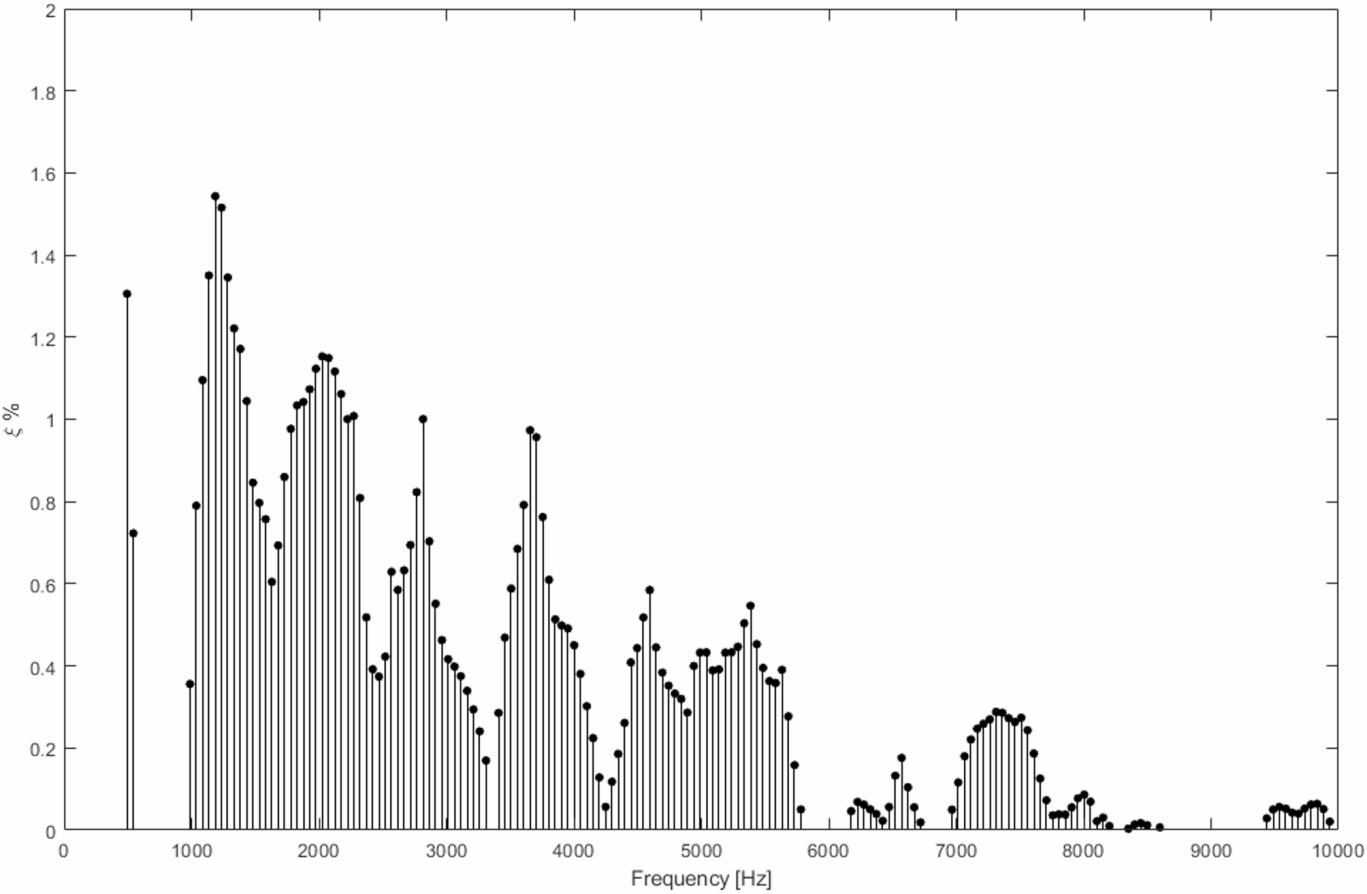
Damping obtained in terms of frequency (central point).

Figure 5 presents the damping results obtained at a central point (near the umbo) on the membrane for different frequencies. In the following section, this analysis will be extended to the entire membrane. Nevertheless, even from this single-point analysis, several interesting observations can be made.

A clear frequency-dependent trend is evident: the damping decreases as the frequency increases. Furthermore, certain frequencies show no measurable damping. It should be noted that the membrane exhibits free vibration only at frequencies close to its natural modes. Outside these frequencies, there is no discernible damped signal to fit, and consequently, no damping value can be extracted. This characteristic implies that the methodology also provides a useful tool to obtain an approximate value for the membrane’s natural frequencies. However, this aspect requires careful interpretation, as the presence and magnitude of each frequency in the measured response are also influenced by the corresponding mode shape. A more detailed discussion of this will be provided in the next section.

In summary, the results obtained so far are consistent, and the proposed methodology is validated. The damping curves can be interpreted as providing an envelope that highlights the frequency regions where the membrane actively responds.

The true potential of this strategy lies in its combination with full-field data acquired through the holographic measurement technique. The availability of displacement data from a large number of points on the membrane significantly enhances the analysis. By performing individual damping assessments at each of these locations, it becomes possible to approximate some natural frequencies excited by the system and to map the corresponding mode shapes in an approximate way, as will be shown in next section.

### Signal processing parameters

The selection of STFT parameters and filtering criteria was guided by a balance between time and frequency resolution. Hanning windows were used, and both window size and number were adjusted to ensure reliable damping estimation.

Several combinations of window number and window size were systematically evaluated to optimize the analysis parameters. The number of windows determines the time resolution of the analysis: increasing the number of windows improves the curve-fitting accuracy and the reliability of the damping estimation. On the other side the size of the window improves the frequency resolution. Both conditions must be balanced to limit the computational cost while keeping sufficient time and frequency resolution.

Hanning windows were employed for the FFT calculations. In particular, within the framework of STFT analysis, window size is a critical parameter that requires careful adjustment to balance frequency and time resolution.

The period of the window means the total duration of each segment being analyzed and limits the lowest frequency you can reliably detect. In this case, the minimum valid frequency is not simply 1/T, but 1.5/T to account for the Hanning window’s effect on measurement accuracy.

The maximum available period of time from the experimental data (20 ms) was used for the analysis. Trial calculations were made with shorter period of time, distributing the windows at the beginning or at the end of the time signal and ignoring the rest. Similar results were obtained. As the magnitude of the signal decreases, the influence of noise at the end of the signal was expected higher in relative terms but no variation has been found when shortening the signal.

Following extensive computational testing to assess the influence of these parameters, the results presented in this work were obtained using 17 Hanning windows, each with a length of 400 points, period of T = 6 ms and fₘ_i_ₙ = 250 Hz. These relatively wide windows enable reliable damping estimation from approximately 500 Hz onwards, while maintaining a reasonable computational cost. The first window is centered at t₁ = 4.4 ms, and the last at t₁₇ = 16.4 ms. The first window is slightly offset from 0.4 ms to ensure that we work on the system’s response under free vibration.

This procedure was applied to the entire measurement grid (256 × 256 points), providing damping values for each spatial location and frequency. Consequently, full-field damping maps of the membrane and umbo regions were obtained.

It must be remarked that the STFT calculation for one single point does not take too much time computation, approximately 1–2 s depending on the computer and number of windows, etc. But the power of this methodology is when we map the entire grid of data obtained from HDH. When we make trials, we used a reduced 64 × 64 points grid, this means 4096 calculations with computing time between 1 and 2 h. The 256 × 256 points grid means 65,536 calculations and it took about 30 h. So, the parameters were established with trials with 64 × 64 data trying to minimize the computational cost for the following calculation.

### Selection criteria

The primary objective of this study is to quantify representative frequency-dependent damping values for the entire middle ear system. Given the great number of data obtained from the measurement matrix, it is possible to compute average damping values at each frequency, thus minimizing potential experimental or numerical errors.

To achieve this, different options have been proposed. One approach is to compute the mean damping value for a given frequency. Another one involves obtaining a weighted damping result based on the excitation level of each point at that frequency. Two specific hypotheses were considered: (1) computing the average damping by considering only the points that exhibit the highest displacement magnitudes for a given vibration mode, or (2) applying a weighting factor to the damping result of each point based on the relative displacement of each point with respect to the maximum displacement.

After evaluating these different procedures, a simple yet robust criterion was adopted: only those points whose displacement amplitude exceeds 80% of the maximum at each frequency are included in the averaging process. This methodology effectively eliminates low-motion regions, which would otherwise distort the final damping estimation, often resulting in artificially low values.

Beyond this analysis, it is also of interest to distinguish between damping mechanics intrinsic to the TM and those associated with other components of the middle ear, such as the ossicular chain. Previous numerical simulations^28^ present better accuracy with this separate damping characterization.

**Fig. 6:**
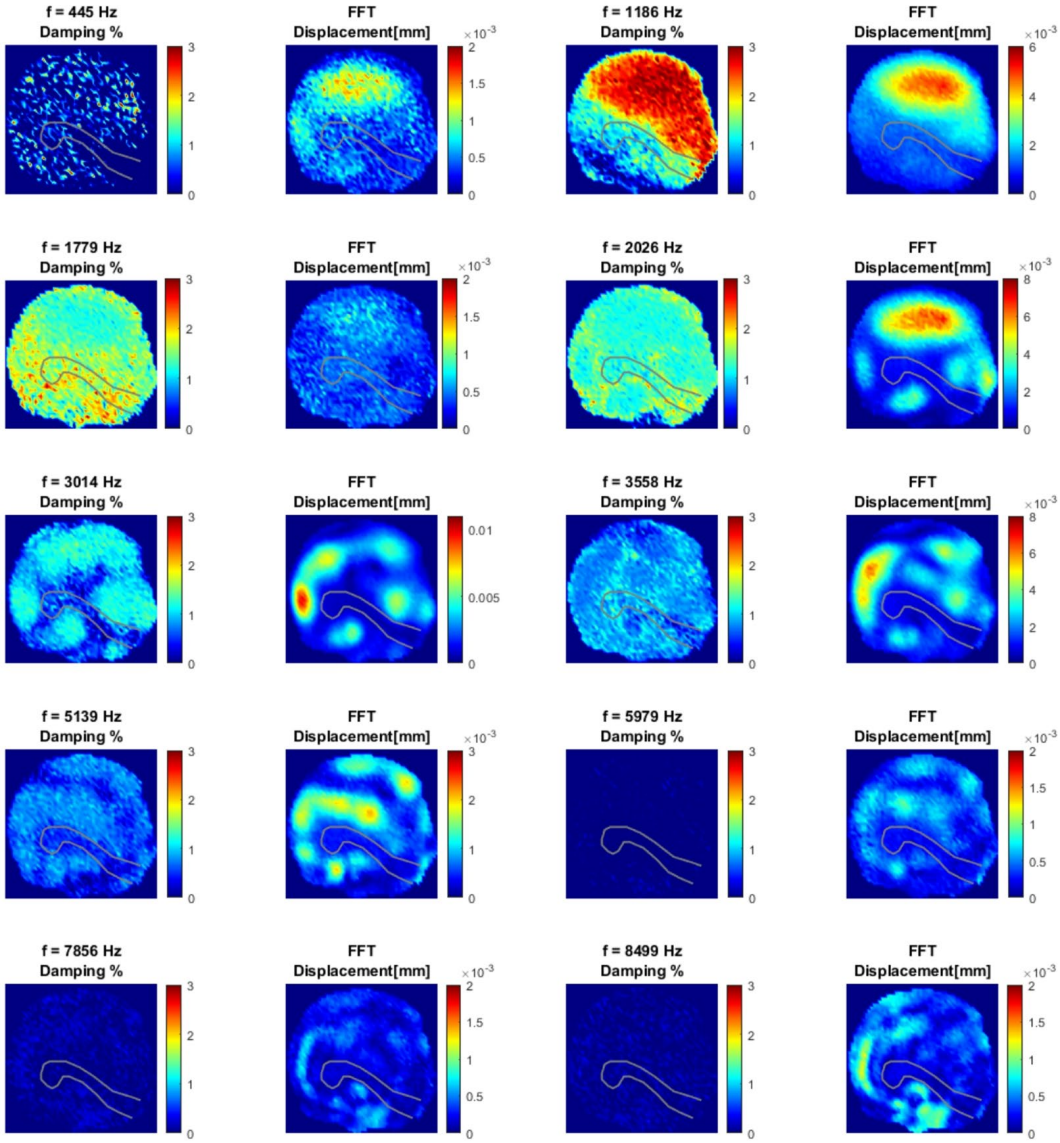
Damping and displacement magnitude maps from FTT analysis at selected frequencies. Note that while a common color scale is used for all damping maps, the displacement magnitude maps are individually scaled for each frequency to highlight modal spatial patterns.

To address this, spatial filtering masks were applied to the dataset to isolate different regions of interest. Three separate masks were defined on the TM surface for comparative analysis: (1) one encompassing the entire TM, including the umbo region (spoon shape outline in Fig. 6) connected to the middle ear ossicles; (2) one isolating the umbo region only; and (3) one covering the remaining area of the membrane while excluding the umbo region.

## Results

Once the methodology described in the previous sections is applied to all points on the TM surface, full-field damping maps can be generated for each analyzed frequency.

**Fig. 7:**
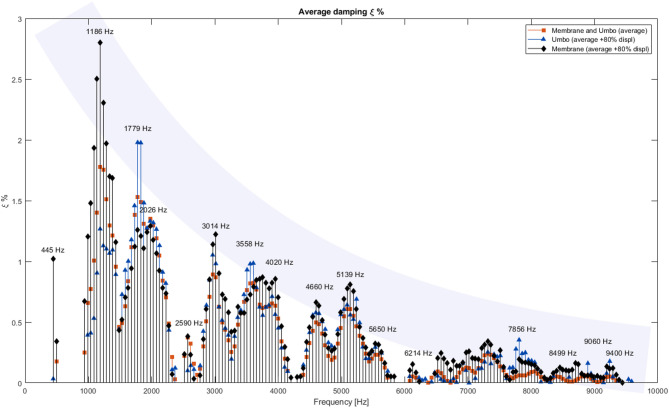
Representative Damping of the system, the umbo and the membrane with frequency.

Figure 6 shows the damping maps for several selected frequencies, alongside the corresponding displacement magnitude maps derived from the FFT of the full measurement matrix. FFT has been calculated from the full time length of 20 ms with a Hanning window. These frequencies were chosen based on several criteria, discussed in detail with reference to Fig. 7. Most of the selected frequencies correspond to the natural frequencies of the membrane system.

In general, a clear correlation is observed between regions of maximum displacement magnitude and maximum damping. This relationship is particularly evident for frequencies such as 1186 Hz, 3014 Hz, and 5139 Hz.

Two primary factors contribute to this observation. The first is related to the mode shapes of the membrane at its natural frequencies. There are areas where no motion is present at that frequency (nodes) and so it is reflected in the magnitude of the displacement. It must be recalled that the presence of nodes (zero displacement location on the mode shape) is not so clear as the magnitude of the response can be affected by adjacent frequencies. The second factor, partially derived from the first, is that regions with higher displacement magnitudes yield more reliable damping estimations. The increased signal in these areas enhances the accuracy and consistency of the curve-fitting process. For instance, the damping map at 1186 Hz shows a homogeneous damping value of approximately 3% in regions where the displacement response is maximal. This observation is highly relevant for establishing a consistent criterion to quantify damping across the system, as will be discussed later.

Nonetheless, this dependence on signal magnitude also represents a limitation, particularly for higher frequencies where the membrane response is weaker. An extreme case is observed at 5979 Hz (note that the displacement color scales vary between plots), where no damping could be reliably estimated, likely due to the absence of natural frequencies in that range. Even at higher frequencies where damping values were obtained (7856 Hz and 8499 Hz), it is probable that these estimations are underestimated due to the low signal magnitude and reduced reliability of the fitting process.

Figure 7 presents the average damping results obtained using each of the proposed criteria from Sect. "Signal processing parameters", plotted as a function of frequency. The red-square results are derived from the entire data matrix representing a global average; however, they are significantly influenced by erroneous damping values originating from regions with minimal displacement, where reliable damping estimation is not feasible. Consequently, these values tend to underestimate the overall damping.

More reliable results are obtained using the 80% displacement threshold criterion. This approach yields more accurate and consistent damping estimations by excluding regions with insufficient motion. Furthermore, two separate average damping curves are provided: one calculated exclusively from the umbo region, reflecting the response of the ossicular chain, and the other excluded the umbo, corresponding to the tympanic membrane’s response only.

The two damping curves obtained—one for the umbo region (blue-triangle) and the other for the membrane (black-diamond)—exhibit similar overall trends but also display significant differences at specific frequencies. The damping curve for the entire system consistently falls between these two. Moreover, the natural frequencies of the system are clearly identifiable as peaks in the damping curves. An approximate broad gray band has been added to the figure to indicate estimated frequency-dependency of damping. It is only for illustrative purposes; no reliable curve fitting can be done at the moment.

By comparing the displacement FFT results shown in Fig. 6 with the natural mode shapes predicted by previous FEM works^28^, a strong correspondence is observed between the experimentally identified natural frequencies and the simulated modes. It is important to emphasize that the frequencies selected for the displacement and damping maps in Fig. 6 were chosen based on the experimental peaks identified in Fig. 7, independently of the numerical simulations^28,50^.

While most of the dominant modes reflect the global response of the entire middle ear system, some are more strongly associated with either the TM or the ossicular chain^28,50^. This distinction becomes evident when examining specific frequencies such as 1779 Hz, 3558 Hz, and 7856 Hz, where the damping measured at the umbo is noticeably higher than that of the membrane. These differences indicate distinct dynamic participation of the umbo and membrane regions at specific frequencies.

For example, at 1779 Hz, the damping in the umbo region reaches approximately 2%, while the membrane exhibits a damping of around 1.4%. The displacement map at this frequency shows relatively uniform motion across both the membrane and the umbo, indicating that the movement is governed by the stiffness and mass of the ossicular chain. This behavior contrasts with the majority of other frequencies, where the membrane exhibits greater motion than the umbo, reflecting its dominant role in the vibration response.

## Discussion

This section focuses on the interpretation of the results presented in Sect. “Results” and their comparison with previous studies. The results obtained by applying the methodology developed in this work represent a significant improvement compared to previous analyses performed on the same dataset. The use of spatial masks, a refined window-based analysis, the filtering of frequencies unrelated to the actual physical behavior of the system, and, most importantly, the preservation of information by avoiding excessive filtering, allow for the identification of a greater number of representative peaks in the Frequency Response Function (FRF) curves compared to earlier results.

From a theoretical standpoint, it must be recalled that in a linear MDOF system the damping ratio associated with a specific mode does not depend on the spatial measurement position. In such systems, damping is defined through the system matrices, and modal damping ratios are intrinsic properties of each vibration mode, independent of the observation point. Therefore, the damping values identified in the present work are not intended to represent intrinsic modal damping ratios of an ideal linear system, but rather experimentally identified local effective damping values obtained from local measurements of the tympanic membrane response.

The proposed methodology is based on full-field transient measurements combined with time–frequency analysis using STFT. Within this framework, the measured response at any spatial location can be expressed as a superposition of multiple vibration modes, each contributing with a different weight depending on the local modal amplitude. Consequently, the identified local effective damping reflects the spatial weighting of the contributing modes, the finite time–frequency resolution of the analysis, and the transient nature of the excitation.

In this context, the spatial differences observed between regions such as the umbo and the membrane do not imply that damping is position-dependent in a strict theoretical sense, nor that the system is non-linear. Instead, they reflect differences in modal participation and local dynamic response within a highly coupled complex system. This interpretation is particularly important for avoiding a misleading association between the reported values and pure modal damping in the theoretical sense.

From a modelling perspective, it is also important to note that, although in a linear MDOF system different damping values can be assigned to each degree of freedom through the damping matrix, numerical models of complex systems such as the middle ear usually rely on simplified damping descriptions, such as proportional damping or reduced parameter sets. This is largely due to the lack of direct experimental information about the contribution of individual dissipation mechanisms. In practice, damping is therefore often introduced as an aggregated or effective property calibrated to reproduce the observed dynamic response. In this regard, one of the main contributions of the present work is to provide spatially resolved experimental evidence of this effective damping, revealing subtle differences in regions such as the umbo. These observations may assist in making more informed decisions when assigning damping parameters to different components of the numerical model, such as the tympanic membrane and the ossicular chain, even if the final model still relies on a reduced representation of the damping matrix.

The methodology provides a framework to estimate local effective damping and has not yet been fully validated in terms of accuracy and precision. Further validation will require its application to a larger set of specimens in order to assess variability and establish general trends applicable to numerical models. At present, the results should be interpreted within the context of the specific specimen analyzed and its preservation state. It should also be noted that, to the best of our knowledge, no experimental techniques with higher spatial and temporal resolution are currently available for direct validation of these measurements.

While qualitative validation is supported by comparisons with previous studies based on HDH and FEM predictions, full quantitative validation in terms of accuracy and precision requires additional experimental datasets. Nevertheless, we consider that this approach allows for a more comprehensive analysis of the extensive dataset obtained through the powerful HDH technique.

**Fig. 8:**
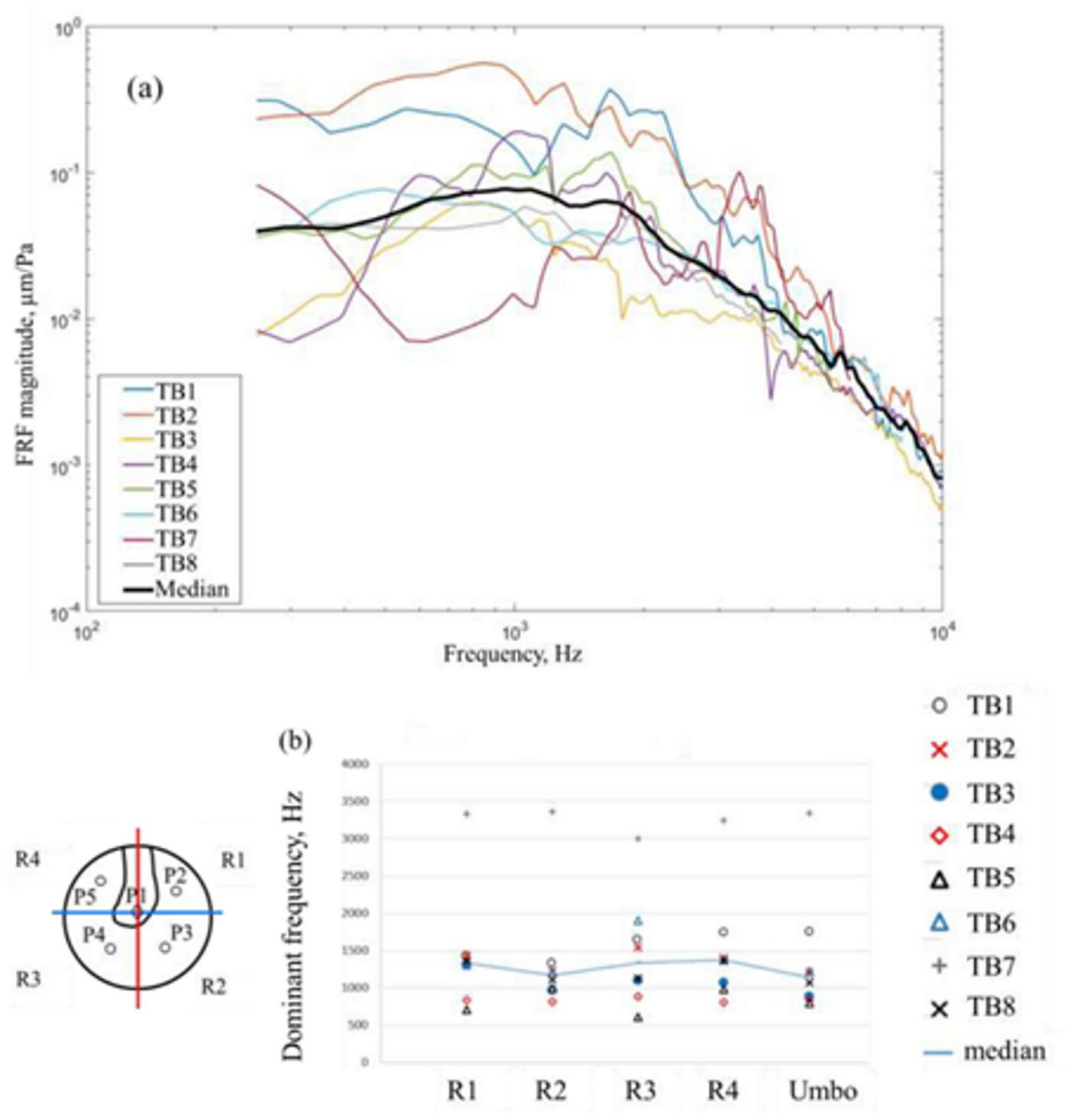
Results from previous works^29^(**a**) Magnitude of the surface averaged FRF of 8 TMs. The median of the 8 measurements is plotted in black. (**b**) four quadrants and umbo averaged value and their median value.

For example, if we examine Fig. 8a, which reproduces the initial HDH data analyses presented in reference^29^, it becomes evident that identifying the natural frequencies of the system was considerably more challenging. Moreover, the global average calculated over the entire membrane surface yielded results that were less interpretable than those obtained using the dedicated umbo and membrane masks presented in this study. Similarly, the dominant frequencies identified for the different specimens are shown (Fig. 8b). These frequencies fall within the same order of magnitude as some of those presented in this work, yet the results exhibited lower precision and frequency resolution.

Finally^29^, reports a preliminary study on the damping ratio, the results exhibit general trends similar to those reported here, such as the decreasing damping with increasing frequency. However, that study provided significantly lower damping values per frequency for each specimen, with large variability in the results and no clear separation between the damping contributions of the umbo and the membrane. In addition, the damping values at higher frequencies appeared underestimated, likely due to information loss caused by excessive filtering applied in that frequency range.

## Conclusions

This work presents a refined and validated methodology for estimating frequency-dependent damping in the human TM based on high-resolution experimental measurements and advanced time-frequency analysis. By combining HDH with STFT techniques, the method enables precise, spatially resolved characterization of TM damping across a broad frequency range.

Damping plays a crucial role in the dynamic behavior of the middle ear (ME) system and remains a complex parameter to quantify experimentally. Accurate estimation of this property is essential to improve FEM used in prosthesis design, pathology diagnosis, and hearing research. Despite advances in measurement techniques and numerical simulations, characterizing TM damping with high spatial and frequency resolution has been a persistent challenge due to limitations in previous methodologies.

The methodology proposed in this study addresses these limitations through several key improvements. The use of HDH allows full-field, high-resolution displacement measurements across the TM surface, capturing transient responses with nanometric precision. The application of STFT to these measurements enables reliable damping estimation at individual frequencies, overcoming the difficulties posed by overlapping vibration modes in the time domain.

A critical aspect is the introduction of spatial filtering masks, which isolate different anatomical regions of the ME, specifically the umbo and the surrounding membrane. This separation provides valuable insight into the respective contributions of the ossicular chain and the TM to the overall damping behavior. Furthermore, the use of a displacement threshold criterion, retaining only points with displacement amplitudes above 80% of the maximum at each frequency, ensures that damping estimates are based on reliable, high-quality data, minimizing distortions from low-motion regions.

The results demonstrate a clear frequency-dependent damping trend, with damping decreasing as frequency increases. In addition, by comparing the average damping curves obtained for the umbo and the membrane, it is possible to distinguish between modes primarily associated with the TM and those influenced by the ossicular chain.

It is important to note that the presented work provides a consistent framework for extracting damping-related information from complex transient responses in systems with multiple degrees of freedom. Compared to previous studies using HDH, it offers significant improvements in the identification of natural frequencies and damping estimation. The refined filtering process, combined with spatial masking, enables the detection of a greater number of representative peaks in the Frequency Response Function (FRF) curves and provides higher precision in both frequency and spatial domains. These results are consistent with previous FEM predictions.

The procedure demonstrates clear advantages and robustness. The present study is based on a single cadaveric specimen. Although the experimental setup and analysis process have been validated, and the obtained results are consistent with those from previous studies, future work should focus on extending this approach to a larger set of specimens. This will allow us for the assessment of inter-subject variability, the identification of potential systematic trends, and the further validation of the procedure across different anatomical configurations.

In conclusion, the methodology developed in this study significantly advances the experimental characterization of TM damping. The method enhances our understanding of the dynamic behavior of the TM and ME system and contributes directly to the development of more accurate simulations and potential clinical applications in hearing restoration and diagnosis.

## Data Availability

The experimental datasets analyzed in this study were obtained at the Eaton-Peabody Laboratory, Massachusetts General Hospital (Boston, USA), and are subject to a Data Use Agreement with the institution. Consequently, the raw data cannot be made publicly available. Access to these datasets may be granted upon reasonable request and subject to approval by the Eaton-Peabody Laboratory. Results obtained in these tests can be consulted in the following references ^19^(doi:10.1016/j.actbio.2023.01.048.), ^22^(doi:10.1016/j.heares.2021.108335) or ^2^(doi:10.3390/app9142809).

## Abbreviations

EC: Ear canal
FEM: Finite element method
FFT: Fast Fourier Transform
HDH: High-speed digital holographic
IRF: Impulse Response Function
MDOF: Multi-degree of freedom
ME: Middle ear
LDV: Laser Doppler vibrometry
OC: Ossicular chain
SPL: Sound Pressure Level
STFT: Short-time Fourier transform
TC: Tympanic cavity
TM: Tympanic membrane
